# The Nod2 Agonist Muramyl Dipeptide Cooperates with the TLR4 Agonist Lipopolysaccharide to Enhance IgG2b Production in Mouse B Cells

**DOI:** 10.1155/2019/2724078

**Published:** 2019-11-26

**Authors:** Sang-Hoon Lee, Jong-Hwan Park, Seok-Rae Park

**Affiliations:** ^1^Department of Microbiology, Myunggok Medical Research Institute, College of Medicine, Konyang University, Daejeon 35365, Republic of Korea; ^2^Laboratory Animal Medicine, College of Veterinary Medicine, Chonnam National University, Gwangju 61186, Republic of Korea; ^3^Priority Research Center, Myunggok Medical Research Institute, College of Medicine, Konyang University, Daejeon 35365, Republic of Korea

## Abstract

Many studies have shown that Toll-like receptors (TLRs) and Nod-like receptors (NLRs) were expressed in B cells and their signaling affects B cell functions. Nonetheless, the roles played by these receptors in B cell antibody (Ab) production have not been completely elucidated. In the present study, we examined the effect of the Nod2 agonist muramyl dipeptide (MDP) in combination with the TLR4 agonist lipopolysaccharide (LPS), a well-known B cell mitogen, on B cell viability, proliferation, and activation, and Ab production by in vitro culture of purified mouse spleen resting B cells. MDP combined with LPS to reinforce B cell viability, proliferation, and activation. Moreover, MDP enhanced LPS-induced IgG2b production, germline *γ*2b transcript (GLT*γ*2b) expression, and surface IgG2b expression. In an experiment with Nod2- and TLR4-deficient mouse B cells, we observed that the combined effect of MDP and LPS is dependent on Nod2 and TLR4 receptors. Furthermore, the combined effect on B cell viability and IgG2b switching was not observed in Rip2-deficient mouse cells. Collectively, this study suggests that Nod2 signaling enhances TLR4-activated B cell proliferation, IgG2b switching, and IgG2b production.

## 1. Introduction

Pattern recognition receptors such as Toll-like receptors (TLRs), Nod-like receptors (NLRs), and C-type lectin receptors (CLRs) recognize specific conserved bacterial structures (pathogen-associated molecular patterns). TLR signaling can directly affect B cell functions, even without the support of T cells [1–4]. We recently reported that TLR1/2 agonist Pam3CSK4 and TLR7 agonist imiquimod directly inhibit IgG1 and IgE class switching, respectively, in activated mouse B cells [5, 6]. In addition, we found that Dectin-1 (a type of CLR) agonist selectively induced IgG1 class switching by TLR4 agonist lipopolysaccharide (LPS)-activated mouse B cells [7, 8]. Many studies have shown that TLR signaling interplays with other receptor signaling such as other TLRs, B cell receptor (BCR), and CD40 signaling in B cells [9–14]. For instance, TLR2 stimulation arrests TLR4 agonist LPS-promoted B cell maturation [15]; BCR signaling synergizes with TLR signaling for activation-induced cytidine deaminase (AID) expression and Ig class switch recombination (CSR) by B cells [16]. Thus, TLRs play various roles in B cell activation, differentiation, and function.

However, the roles played by NLRs (Nod1, Nod2, NLRC4, NLRP3, etc.) in B cells remain to be elucidated. The effects of Nod1 and Nod2 stimulation on B cell activation have been investigated in only a few studies: Cohen and Parant reported that Nod2 agonist muramyl dipeptide (MDP) increases surface Ig (membrane *κ*-light chain) expression and enhances the response to LPS in the mouse pre-B cell line 70Z/3 [17]; Petterson et al. reported that Nod1 or Nod2 stimulation augments BCR- or TLR-induced human B cell activation (proliferation, viability, and expression of cell surface markers) independently of physical T cell assistance [18, 19].

In the present study, to further elucidate the role of Nod2 in B cell response, we directly stimulated mouse resting B cells with MDP in the absence and presence of LPS in vitro and analyzed B cell viability, proliferation, activation, antibody (Ab) production, and Ig class switching.

## 2. Materials and Methods

### 2.1. Animals

Wild-type (WT) C57BL/6 mice were purchased from Damool Science (Daejeon, Korea). TLR4-deficient (*Tlr4^−/−^*), Nod2-deficient (*Nod2^−/−^*), and Rip2-deficient (*Rip2^−/−^*) mice with a C57BL/6 background were purchased from Jackson Laboratory (Bar Harbor, ME, USA). Mice were maintained on an 8 : 16 h light:dark cycle in an animal environmental control chamber. Eight- to twelve-week-old mice were used, and animal care was provided in accordance with the guidelines of the Institutional Animal Care and Use Committee of Konyang University.

### 2.2. Cell Culture and Reagents

The mouse B cell lines L10A6.2 (surface *μ*^+^, mature B cell line) and A20.3 (surface *γ*2a^+^) were provided by Dr. J. Stavnezer (University of Massachusetts Medical School, Worcester, MA, USA). The mouse B lymphoma cell line CH12F3-2A (surface *μ*^+^) was provided by Dr. T. Honjo (Kyoto University, Kyoto, Japan). Mouse spleen resting B cells were obtained by depletion of CD43^+^ cells using anti-CD43 microbeads and high-gradient magnetic cell separation according to the manufacturer's instruction (MACS; Miltenyi Biotec, Bergisch Gladbach, Germany) as previously described [5]. The purity of resting B cells (CD43^−^B220^+^) was assessed using FACSCalibur (BD Biosciences, San Jose, CA, USA) following staining of the cells with anti-CD43 FITC (eBioscience, San Diego, CA, USA) and anti-B220 PE (BD Biosciences) (Supplementary [Supplementary-material supplementary-material-1]). The cells were cultured at 37°C in a humidified CO_2_ incubator (Forma Scientific, Marietta, OH, USA) in RPMI-1640 medium (Welgene, Daegu, Korea) supplemented with 10% fetal bovine serum (PAA Laboratories, Etobicoke, ON, Canada). The cells were stimulated with LPS (ultrapure LPS, *E. coli* 0111:B4; InvivoGen, San Diego, CA, USA), MDP (InvivoGen), and iE-DAP (InvivoGen). The mouse macrophage cell line RAW264.7 was cultured in DMEM (Welgene) containing 2 mM L-glutamine, 100 U/mL penicillin, 100 *μ*g/mL streptomycin, and 10% fetal bovine serum in a humidified CO_2_ incubator. Anti-mouse IgG2b-PE and anti-mouse IgG3-PE were purchased from Southern Biotech (Birmingham, AL, USA). Anti-mouse IgM-PE was obtained from eBioscience.

### 2.3. Cell Viability, Proliferation, and Activation Assays

Cell viability was determined using the EZ-Cytox cell viability assay (DaeilLab Service Co., Ltd., Seoul, Korea) according to the manufacturer's instructions [8]. For the cell proliferation assay, purified mouse resting B cells were labeled with CFSE (eBioscience) and then supplemented with MDP, iE-DAP, and LPS. CFSE dilution was measured by counting 10,000 cells with the FACSCalibur. For the cell activation assay, cultured cells were stained with anti-CD69-FITC (BD Biosciences), and the expression levels were analyzed by flow cytometry (FACSCalibur).

### 2.4. Isotype-Specific ELISAs

Antibodies produced in B cell cultures were detected using isotype-specific ELISAs as previously described [8].

### 2.5. RNA Isolation and RT-PCR

RNA isolation and RT-PCR were performed as previously described [6]. The PCR primers (Supplementary [Supplementary-material supplementary-material-1]) were synthesized by Bioneer (Daejeon, Korea). PCR for *β*-actin was performed in parallel to normalize for cDNA concentrations within each set of samples. PCR products were resolved by electrophoresis on 2% agarose gels. Semiquantitative RT-PCR analysis was performed using cDNA dilutions.

### 2.6. Flow Cytometric Analysis

Surface staining was performed with anti-mouse IgG2b-PE, anti-mouse IgG3-PE, or anti-mouse IgM-PE in the dark for 30 min at 4°C, and surface Ig-expressing B cells were analyzed by flow cytometry (FACSCalibur). Dead cells were excluded from analysis using Zombie Red™ Fixable Viability Kit according to the manufacturer's instruction (BioLegend, San Diego, CA).

### 2.7. Statistical Analysis

Statistical differences between experimental groups were determined by analysis of variances. All *p* values were calculated using unpaired 2-tailed Student's *t*-tests to assess statistical significance.

## 3. Results and Discussion

### 3.1. Dosage Effect of Nod2 Agonist MDP on B Cell Viability and Proliferation

**Figure 1:**
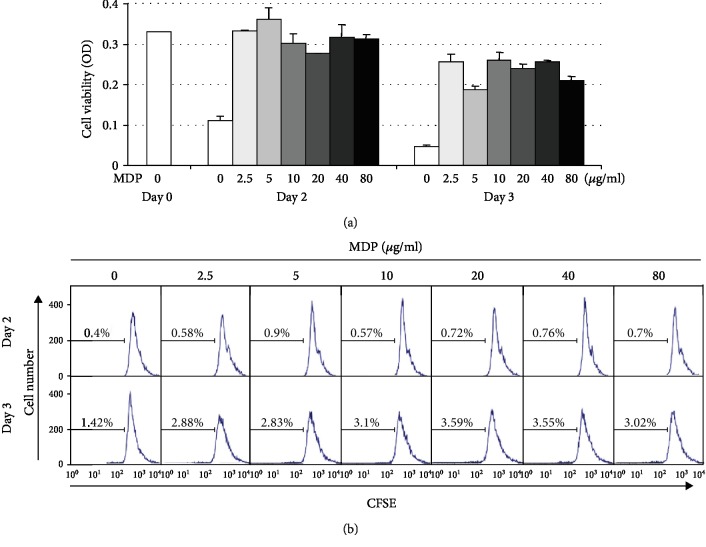
MDP sustains B cell viability in vitro, but MDP itself hardly induces B cell proliferation. Resting B cells were stimulated with the indicated MDP concentrations. After 2 and 3 days of culture, cell viability was measured by (a) EZ-Cytox assay and (b) cell proliferation was measured by CFSE assay. Data shown are representative of two independent experiments.

First, to determine the direct effect of the Nod2 agonist MDP on B cell viability and proliferation, we purified resting B cells from mouse spleen (Supplementary [Supplementary-material supplementary-material-1]) and treated them with MDP. The resting B cells expressed Nod1 and Nod2 as well as TLR4 (Supplementary [Supplementary-material supplementary-material-1]). The RAW264.7 mouse macrophage cell line was used as a positive control for TLR and NLR expression. Resting B cells could not survive in the absence of stimuli in vitro and died (Figure 1(a), white bars). MDP treatment sustained B cell viability but did not increase it in a dose-dependent manner. MDP very slightly induced B cell proliferation (Figure 1(b)). However, MDP alone did not induce any Ab production (data not shown). These results suggest that MDP sustains B cell viability, but MDP itself hardly induces B cell proliferation and plasma cell differentiation.

### 3.2. Nod2 Agonist MDP but Not Nod1 Agonist iE-DAP Combines with TLR4 Agonist LPS to Induce B Cell Viability and Proliferation and IgG2b Production

**Figure 2:**
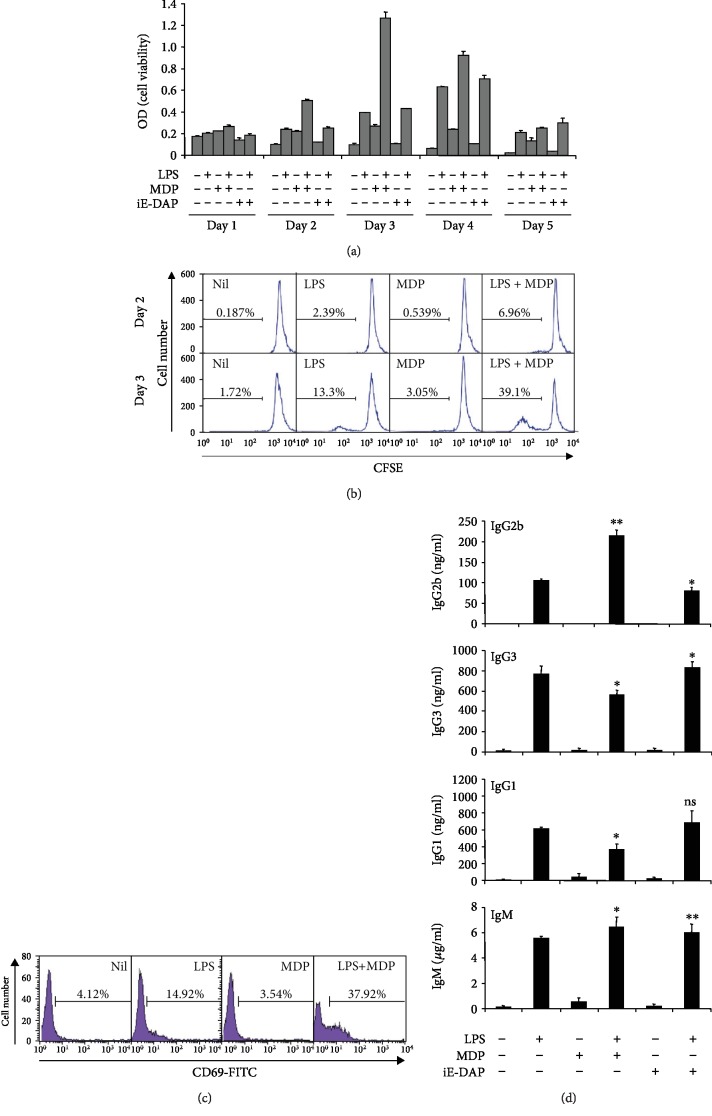
Combined effects of LPS and MDP on B cell viability, proliferation, activation, and Ab production. Resting B cells were stimulated with MDP (10 *μ*g/mL) or iE-DAP (10 *μ*g/mL) in the presence or absence of LPS (1 *μ*g/mL). (a) Cell viability was measured by EZ-Cytox assay at the indicated time points (days). Data presented are means of duplicate samples with ranges (bars). (b) After 2 and 3 days of culture, cell proliferation was measured using CFSE assay. (c) After 2 days of culture, B cell activation was determined by surface CD69 expression. (d) After 7 days of culture, supernatants were harvested, and the levels of Ab production were measured using isotype-specific ELISA. Data presented are the means ± SEM from three independent experiments. ^∗^*p* < 0.05, ^∗∗^*p* < 0.01, SEM: standard error of the mean; ns: not significant.

New functions in innate immune cells have been reported for the crosstalk between TLRs and NLRs [20–22]. There is a synergistic stimulation of human monocytes and dendritic cells by TLR4 and Nod1- and Nod2-activating agonists [23]. Furthermore, Nod2 is involved in TLR4-mediated signaling of inflammation regulation [24, 25]. LPS stimulates TLR4 and is a well-known mitogen for mouse B cells [26, 27]. TLR4 on B cells recognizes LPS and stimulates B cell proliferation, differentiation, and Ig CSR. LPS in vitro stimulation increases IgG2b and IgG3 production through IgG2b and IgG3 class switching, respectively, by mouse B cells [4, 28–33]. Therefore, we investigated the combined effect of TLR4 agonist LPS and Nod2 agonist MDP or Nod1 agonist iE-DAP on B cell viability, proliferation, activation, and Ab production. Resting B cells were stimulated with MDP or iE-DAP in the presence or absence of LPS. After 2 and 3 days of culture, LPS-induced cell viability was significantly enhanced by MDP but not by iE-DAP (Figure 2(a)). In addition, MDP reinforced LPS-induced cell proliferation (Figure 2(b)). These results indicate that MDP combined with LPS to induce B cell viability and proliferation, while iE-DAP does not. Further, MDP enhanced LPS-induced expression of CD69, which is an activation marker (Figure 2(c)). Next, we examined the effect of MDP on LPS-induced Ab production, particularly IgG2b and IgG3 production. MDP increased LPS-induced IgG2b production but decreased LPS-induced IgG3 and IgG1 production (Figure 2(d)). iE-DAP had no significant effect on LPS-induced Ab production. Instead, iE-DAP decreased LPS-induced IgG2b production. These results indicate that MDP combines with LPS to selectively induce IgG2b production.

### 3.3. MDP Combines with LPS to Induce Germline *γ*2b Transcripts and Surface IgG2b Expression

**Figure 3:**
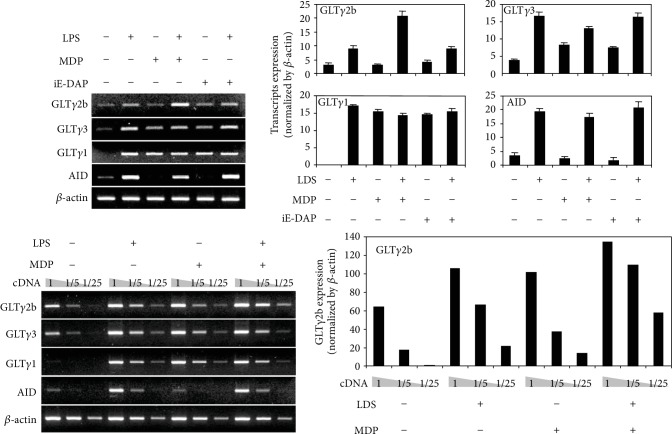
Combined effects of LPS and MDP on the expression of germline transcripts and AID mRNA. Resting B cells were stimulated with MDP (10 *μ*g/mL) and iE-DAP (10 *μ*g/mL) in the presence or absence of LPS (1 *μ*g/mL). After 2.5 days of culture, RNAs were isolated, and the levels of germline transcripts and AID mRNA were measured by RT-PCR. The levels of germline transcripts and AID mRNA were measured by semiquantitative RT-PCR with 1/5 and 1/25 diluted cDNA (lower panel). The graphs show relative transcript levels normalized to the expression of *β*-actin cDNA by ImageJ (NIH, Bethesda, MD, USA) analysis. Densitometric data are averages of two independent experiments with ranges (bars).

**Figure 4:**
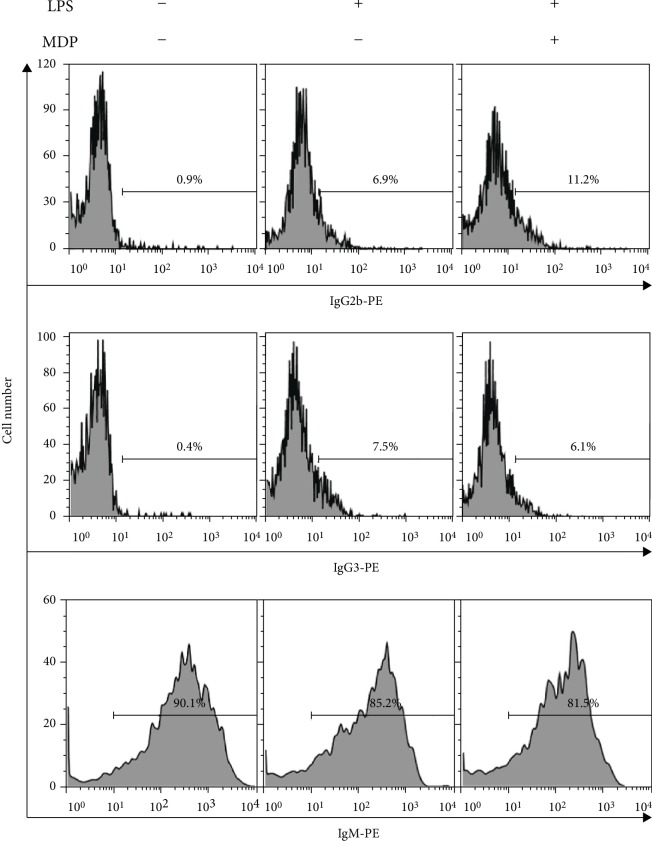
Combined effects of LPS and MDP on surface Ig expression. Resting B cells were stimulated with LPS (1 *μ*g/mL) and MDP (10 *μ*g/mL). After 4 days of culture, cells were stained with anti-IgG2b-PE, anti-IgG3-PE, or anti-IgM-PE, and surface Ig expression was analyzed by flow cytometry.

The transcription of germline transcripts (GLT) is a prerequisite for subsequent Ig CSR [34–36]. Therefore, GLT expression can serve as a marker of Ig class switching. LPS induces the expression of GLT*γ*2b as well as that of GLT*γ*3 [34, 37, 38]. To evaluate the effect of MDP on LPS-induced IgG2b class switching, we examined whether LPS and MDP together induce the expression of germline *γ*2b transcripts (GLT*γ*2b) and surface IgG2b. Resting B cells were stimulated with MDP and iE-DAP in the presence or absence of LPS, and GLT expression were measured by RT-PCR (Figure 3). LPS-induced GLT*γ*2b expression was enhanced by MDP, whereas MDP did not affect LPS-induced GLT*γ*3 and GLT*γ*1 expression (Figure 3). In contrast, iE-DAP neither had any effect on LPS-induced GLT*γ*2b expression nor on GLT*γ*3 and GLT*γ*1 expression. Because AID is an essential enzyme for class switching [39], we assessed its expression. MDP did not affect LPS-induced AID mRNA expression (Figure 3). In addition, MDP selectively enhanced LPS-induced surface IgG2b expression (Figure 4). MDP alone did not induce surface IgG2b expression (data not shown). Collectively, these results indicate that LPS and MDP together induce IgG2b production through increasing IgG2b class switching.

### 3.4. Combination Effect of LPS and MDP Is Abrogated in TLR4- and Nod2-Deficient B Cells

**Figure 5:**
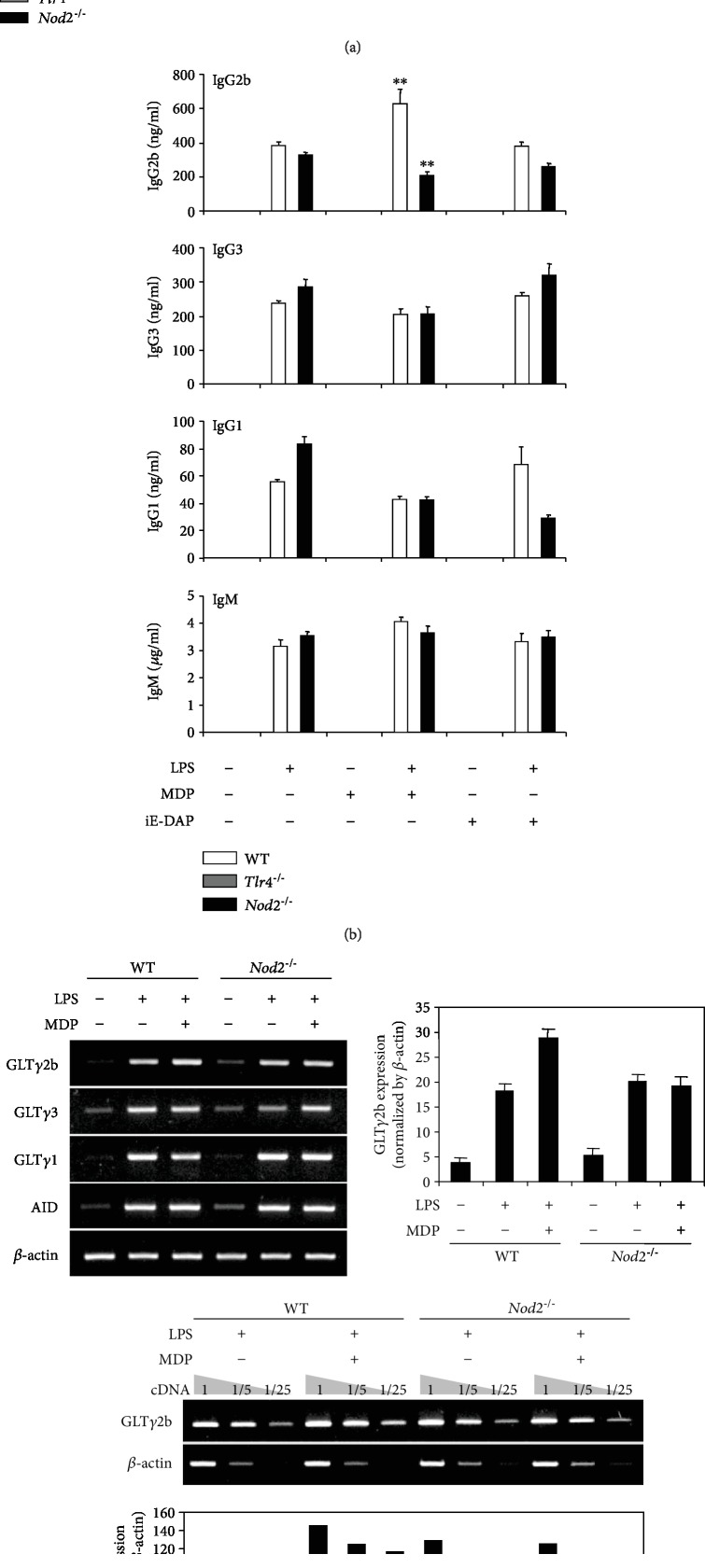
Effects of LPS and MDP on cell viability, proliferation, and Ab production and germline transcripts expression in TLR4- and Nod2-deficient B cells. Resting B cells were purified from wild-type (WT), TLR4-deficient (*Tlr4^−/−^*), and Nod2-deficient (*Nod2^−/−^*) B cells and stimulated with MDP (10 *μ*g/mL) and iE-DAP (10 *μ*g/mL) in the presence or absence of LPS (1 *μ*g/mL). (a) After 2 and 3 days of culture, cell viability (OD) was measured by EZ-Cytox assay kit, and cell proliferation was measured by CFSE assay. Low CFSE intensity cell (%) means the proportion of proliferating cells. (b) After 7 days of culture, supernatants were harvested, and the levels of Ab production were measured by isotype-specific ELISA. Data shown are averages of triplicate cultures with SEM error bars. SEM: standard error of the mean. ^∗∗^*p* < 0.01. (c) After 2.5 days of culture, RNAs were isolated and the levels of germline transcripts and AID mRNA were measured by RT-PCR. The levels of germline transcripts and AID mRNA were measured by semiquantitative RT-PCR with 1/5 and 1/25 diluted cDNA (c, lower panel). The graphs show relative GLT*γ*2b level normalized to *β*-actin cDNA expression using ImageJ, and data are averages of two independent experiments with ranges (bars).

**Figure 6:**
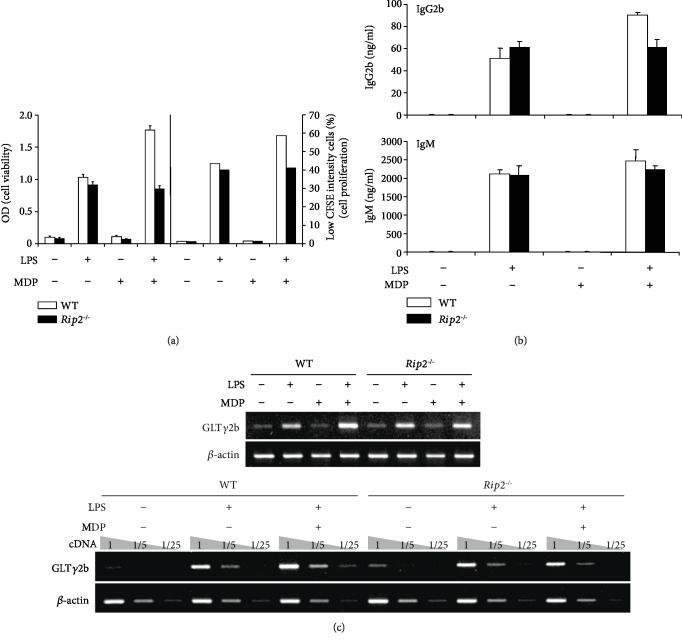
Effects of LPS and MDP on cell viability, cell proliferation, IgG2b production, and germline *γ*2b transcripts expression in Rip2-deficient B cells. Resting B cells were purified from WT and Rip2-deficient (*Rip2^−/−^*) B cells and stimulated with MDP (10 *μ*g/mL) and LPS (1 *μ*g/mL). (a) After 2 and 3 days of culture, cell viability (OD) and proliferation were measured by EZ-Cytox assay and CFSE assay, respectively. Low CFSE intensity cell (%) means the proportion of proliferating cells. (b) After 7 days of culture, supernatants were harvested and the levels of Ab production were measured using isotype-specific ELISA. Data shown are averages of triplicate cultures with SEM error bars. SEM: standard error of the mean. (c) After 2.5 days of culture, RNAs were isolated and the levels of germline *γ*2b transcripts were measured by RT-PCR. The levels of germline *γ*2b transcripts were measured by semiquantitative RT-PCR with 1/5 and 1/25 diluted cDNA (c, lower panel).

LPS and MDP are specific agonists for TLR4 and Nod2, respectively. Therefore, we examined whether the effects of LPS and MDP on B cell responses are dependent on their specific receptors by comparing B cells from WT and TLR4-deficient (*Tlr4^−/−^*) or Nod2-deficient (*Nod2^−/−^*) mice. WT, TLR4-, and Nod2-deficient B cells were stimulated with MDP or iE-DAP in the presence or absence of LPS, and cell viability, cell proliferation, and Ab production were measured (Figure 5). In TLR4-deficient B cells, LPS did not induce cell viability and proliferation (Figure 5(a), gray bars). This finding confirms that B cell proliferation by LPS is dependent on TLR4. In Nod2-deficient B cells, MDP did not enhance LPS-induced cell viability and proliferation (Figure 5(a), black bars). This indicates that MDP can enhance LPS-induced B cell proliferation through Nod2. Next, we examined the effects of LPS and MDP on Ab production in TLR4- and Nod2-deficient B cells. TLR4-deficient B cells did not produce all Abs production upon stimulation of LPS (Figure 5(b)). In Nod2-deficient B cells, MDP did not increase LPS-induced IgG2b production. Furthermore, MDP did not increase LPS-induced GLT*γ*2b expression in Nod2-deficient B cells (Figure 5(c)). These results suggest that the combined effect of LPS and MDP on B cell proliferation and IgG2b production is dependent on their receptors, TLR4 and Nod2. In addition, we investigated the effects of LPS and MDP on B cell responses in receptor-interacting protein 2 (Rip2)-deficient (*Rip2^−/−^*) B cells (Figure 6), because Rip2 is a critical mediator of Nod2 signaling in innate and adaptive immune responses [40–43]. MDP neither reinforced LPS-induced cell viability nor increased cell proliferation (Figure 6(a)), IgG2b production (Figure 6(b)), or GLT*γ*2b expression (Figure 6(c)) in Rip2-deficient B cells. Thus, Nod2-Rip2-mediated signaling could cooperatively play a critical role in LPS-induced B cell responses. However, the underlying molecular mechanisms remain to be determined.

## 4. Conclusions

Our present observations demonstrate that direct stimulation of Nod2 selectively enhances TLR4 agonist LPS-induced IgG2b production by enhancing IgG2b class switching in mouse B cells. IgG2b is particularly important early in the immune response, when T cell support may be limited (i.e., T-independent response), and provides early Fc*γ*R-mediated effector functions and efficient complement activation through binding on C1q [31, 44–46]. Consequently, Nod2 agonist MDP can be used as B cell adjuvant to protect from fast-replicating bacterial infection through enhancing direct B cell activation and IgG2b production independent of T cells and BCR stimulation.

## Data Availability

All data supporting the findings of this study, including its supplementary information files, are available from the corresponding author upon reasonable request.

## Abbreviations

TLR: Toll-like receptor
NLR: Nod-like receptor
LPS: Lipopolysaccharide
MDP: Muramyl dipeptide
Ab: Antibody
GLT: Germline transcripts
CSR: Class switch recombination.

## Supplementary Materials

Supplementary Materials Supplementary Table 1: RT-PCR primers. Supplementary Figure 1: Purity of resting B cells and expression of TLR4, Nod1, and Nod2 in the resting B cells. (a) Purity of isolated mouse spleen resting B cells (CD43^−^B220^+^) was measured using flow cytometric analysis. (b) Total RNA was isolated from the resting B cells and the indicated cell lines. The levels of TLR4, Nod1, and Nod2 mRNA were measured by RT-PCR.
