# Supplementary material for: The Nod2 Agonist Muramyl Dipeptide Cooperates with the TLR4 Agonist Lipopolysaccharide to Enhance IgG2b Production in Mouse B Cells
Source: J Immunol Res. 2019 Nov 26;2019:2724078. doi: 10.1155/2019/2724078 (PMC6899285; doi:10.1155/2019/2724078)
Supplement: Supplementary Materials — Supplementary Table 1: RT-PCR primers. Supplementary Figure 1: Purity of resting B cells and expression of TLR4, Nod1, and Nod2 in the resting B cells. (a) Purity of isolated mouse spleen resting B cells (CD43−B220+) was measured using flow cytometric analysis. (b) Total RNA was isolated from the resting B cells and the indicated cell lines. The levels of TLR4, Nod1, and Nod2 mRNA were measured by RT-PCR. [file 2724078.f1.pdf]

Supplementary Materials

SUPPLEMENTARY TABLE 1: RT-PCR primers

Primer (product size)	Sequence
TLR4 (249 bp)	Forward: 5′-CTGGGTGAGAAATGAGCTGG-3′<br>Reverse: 5′-CTGGGTGAGAAATGAGCTGG-3′
Nod1 (547 bp)	Forward: 5′-AGCTGCAGCCTTGCTTTAGCC-3′<br>Reverse: 5′-TCAGCCATAAATGCCGTAGCG-3′
Nod2 (476 bp)	Forward: 5′-CCGAAGCCCTAGCACTGATGC-3′<br>Reverse: 5′-CAACCATCACGACTCCTCGGG-3′
GLTγ2b (318 bp)	Forward: 5′-GGGAGAGCACTGGGCCTT-3′<br>Reverse: 5′-AGTCACTGACTCAGGGAA-3′
GLTγ3 (349 bp)	Forward: 5′-CAAGTGGATCTGAACACA-3′<br>Reverse: 5′-GGCTCCATAGTTCCATT-3′
GLTγ1 (532 bp)	Forward: 5′-CAGCCTGGTGTCAACTAG-3′<br>Reverse: 5′-CTGTACATATGCAAGGCT-3′
AID (405 bp)	Forward: 5’-CCTCCTGCTCACTGGACTTC-3′<br>Reverse: 5’-GTCTGGTTAGCCGGACAGAA-3′
β-actin (318 bp)	Forward: 5′-CATGTTTGAGACCTTCAACACCCC-3′<br>Reverse: 5′-GCCATCTCCTGCTCGAAGTCTAG-3′

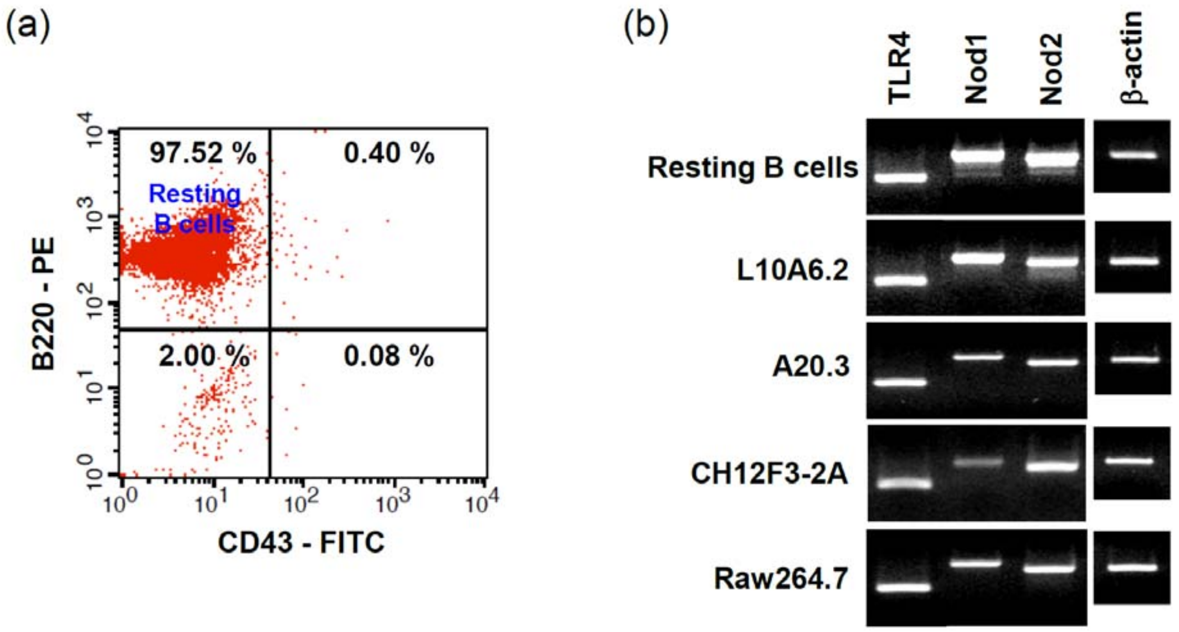

SUPPLEMENTARY FIGURE 1: Purity of resting B cells and expression of TLR4, Nod1, and Nod2 in the resting B cells. (a) Purity of isolated mouse spleen resting B cells ($CD43^-$ $B220^+$) was measured using flow cytometric analysis. (b) Total RNA was isolated from the resting B cells and the indicated cell lines. The levels of TLR4, Nod1, and Nod2 mRNA were measured by RT-PCR.
